# Hemagglutinin 222D/G Polymorphism Facilitates Fast Intra-Host Evolution of Pandemic (H1N1) 2009 Influenza A Viruses

**DOI:** 10.1371/journal.pone.0104233

**Published:** 2014-08-27

**Authors:** Nora Seidel, Andreas Sauerbrei, Peter Wutzler, Michaela Schmidtke

**Affiliations:** Jena University Hospital, Friedrich Schiller University Jena, Department of Virology and Antiviral Therapy, Jena, Germany; University of Georgia, United States of America

## Abstract

The amino acid substitution of aspartic acid to glycine in hemagglutinin (HA) in position 222 (HA-D222G) as well as HA-222D/G polymorphism of pandemic (H1N1) 2009 influenza viruses (A(H1N1)pdm09) were frequently reported in severe influenza in humans and mice. Their impact on viral pathogenicity and the course of influenza has been discussed controversially and the underlying mechanism remained unclarified. In the present study, BALB/c mice, infected with the once mouse lung- and cell-passaged A(H1N1)pdm09 isolate A/Jena/5258/09 (mpJena/5258), developed severe pneumonia. From day 2 to 3 or 4 post infection (p.i.) symptoms (body weight loss and clinical score) continuously worsened. After a short disease stagnation or even recovery phase in most mice, severity of disease further increased on days 6 and 7 p.i. Thereafter, surviving mice recovered. A 45 times higher virus titer maximum in the lung than in the trachea on day 2 p.i. and significantly higher tracheal virus titers compared to lung on day 6 p.i. indicated changes in the organ tropism during infection. Sequence analysis revealed an HA-222D/G polymorphism. HA-D222 and HA-G222 variants co-circulated in lung and trachea. Whereas, HA-D222 variant predominated in the lung, HA-G222 became the major variant in the trachea after day 4 p.i. This was accompanied by lower neutralizing antibody titers and broader receptor recognition including terminal sialic acid α-2,3-linked galactose, which is abundant on mouse trachea epithelial cells. Plaque-purified HA-G222-mpJena/5258 virus induced severe influenza with maximum symptom on day 6 p.i. These results demonstrated for the first time that HA-222D/G quasispecies of A(H1N1)pdm09 caused severe biphasic influenza because of fast viral intra-host evolution, which enabled partial antibody escape and minor changes in receptor binding.

## Introduction

In 2009, a new pandemic (H1N1) influenza virus (A(H1N1)pdm09) emerged in Mexico and spread around the world, causing the first pandemic since decades [1]. The genome of A(H1N1)pdm09 virus comprises six gene segments originating from North American triple-reassortant swine viruses and two, the neuraminidase (NA) and the matrix gene segments, from Eurasian swine influenza A viruses [1]. In the majority of cases, A(H1N1)pdm09 virus infection was associated with mild disease [2]. But, there were cases of severe and fatal outcome observed in young healthy adults and pregnant women [3]–[6].

Amino acid substitution of aspartic acid to glycine at position 222 (D222G; H1 numbering) in the hemagglutinin (HA-D222G) was supposed to be associated with severe influenza and fatality in humans [7]–[11], although this substitution was also detected in virus isolates from patients with mild influenza [12], [13]. The HA-D222G occurred among clinical isolates during growth of the viruses in the laboratory [14] as well as after passaging A(H1N1)pdm09 in embryonated chicken eggs [15]. In addition, HA-222D/G polymorphism of A(H1N1)pdm09 has been described in humans [7]–[10], [13], [16]. According to Wedde et al. [16], the pure HA-G222 variant, HA-222D/G, HA-222D/G/N, and HA-222D/G/N/V/Y are co-circulating in hospitalized patients with severe influenza and fatal outcome. The amino acid substitution of HA-D222G was also detected during the adaptation of A(H1N1)pdm09 viruses to mice [17]–[20]. It was described by many as a virulence increasing determinant [15], [19]–[21], but not confirmed by all [22]. Thus, despite frequent description of the amino acid substitution HA-D222G and HA-222D/G polymorphism, the impact of these quasispecies on the course of influenza was discussed rather controversially until now.

HA-222 is part of the Ca2 antigenic site [23] as well as the receptor binding site [24]. A carbohydrate microarray analysis revealed that A(H1N1)pdm09 viruses have broad specificity for both α-2,3- and α-2,6-linked sialic acid receptors, but the binding affinity towards a considerable range of α-2,3-linked sialyl sequences is generally lower than to their α-2,6-linked counterpart [25]. Both HA-D222 and HA-G222 isolates of A(H1N1)pdm09 preferentially bind terminal sialic acid (SA) α-2,6-linked to galactose (SAα2-6Gal), the human influenza receptor, whereas HA-G222 variants were shown to have higher affinity to SAα2-3Gal than HA-D222 variants [22], [26], [27]. This dual receptor binding specificity of the A(H1N1)pdm09 HA enables the viruses to infect hosts with different receptors e.g. human, swine and mice, without major change.

Some A(H1N1)pdm09 isolates caused severe influenza with biphasic body weight loss in mice [11], [18], [28], [29]. The reason behind was either not explained by the authors or simply deduced as certain immune response. To unveil the mechanism underneath the biphasic course of severe influenza caused by A(H1N1)pdm09, BALB/c mice were infected with the A(H1N1)pdm09 isolate mpJena/5258 and symptoms of disease (body weight, clinical score) were monitored over 12 days daily. Lung, trachea, and serum samples of virus-infected mice were collected daily from day 1 till day 7, on day 9, and day 12 p.i. Analysis of lung histopathology, virus replication in lung and trachea, viral intra-host evolution, as well as the host neutralizing antibody response indicated the impact of HA-222D/G polymorphism in combination with host factors on influenza virus adaption.

## Materials and Methods

### Ethics statement

All trial procedures and animal care activities were conducted in accordance with the German Animal Protection Law. Experiments were approved by the Thüringer Landesamt für Verbraucherschutz (Reference Numbers: 02-049/11 and 02-032/12).

### Cells and viruses

Madin-Darby canine kidney (MDCK) cells (Friedrich-Loeffler Institute, Riems, Germany) and Madin-Darby bovine kidney (MDBK) cells (IDT Biologika GmbH, Dessau-Rosslau, Germany) were maintained in Eagle's minimum essential medium (EMEM) supplemented with 100 U/ml penicillin and streptomycin, 10% fetal bovine serum, and 2 mM L-glutamine. All tests and virus propagation were performed with EMEM formulated with 100 U/ml penicillin and streptomycin, 2 µg/ml trypsin, and 0.1% sodium bicarbonate (test medium).

After one passage of Jena/5258 [29], [30] in a mouse lung, the virus was propagated in MDCK cells to get a stock (mpJena/5258) for *in vivo* experiments. The amino acid sequences of the eight gene segments of the original and the mouse-passaged isolate were identical (GenBank accession numbers KJ549775–KJ549782).

The HA-D222-mpJena/5258 virus was isolated by three serial plaque purification steps from mpJena/5258. The homogenized trachea of an mpJena/5258-infected mouse dissected on day 5 p.i. was used to isolate the HA-G222-mpJena/5258 virus by three serial plaque purification steps.

### Determination of virus titer

Virus titers were determined by titration of 10-fold serial dilutions of virus suspensions (each 4 parallels) on confluent MDCK cells in test medium. The 50% tissue culture infectious dose (TCID_50_) was calculated according to Reed and Muench [31].

### Plaque purification

Plaque purification of viruses was performed as described previously [32]. In brief, confluent MDCK cells in 12-well plates were incubated with 10-fold serial dilutions of the virus for one hour. The infection medium was removed and cells were overlaid with test medium containing 0.4% agar. After 48 hours of incubation at 37°C single plaques were picked and two further rounds of plaque purifications were performed. Picked viruses were propagated on MDCK cells to get a virus stock for further studies. Sequencing of the complete HA was used to compare the plaque-purified virus with the original isolate.

### Sequencing

Total RNA was isolated from the supernatants of infected MDCK cells or from organ homogenates with the RNeasy Mini Kit (Qiagen, Hilden, Germany) according to the manufacturer's instructions. Virus specific RNA was amplified by reverse transcription using the Omniscript RT Kit (Qiagen, Hilden, Germany) and the Uni 12 primer [33] according to the manufacturer's protocol. Amplification of viral gene segments was performed using the Taq Core Kit 10 (MP Biomedicals, Eschwege, Germany) and the segment specific primers described by Hoffmann et al. [33]. Conditions of the PCR, purification of PCR products and sequencing were described previously [32]. Sequencing was carried out with the segment specific primers [33] and A(H1N1)pdm09-specific primers 5′-CTTTCCAYCATGTATGCCACCAT-3′, 5′-TGTTATCATGGAGGTCGTTTTCCC-3′, 5′-CTGTTCGTCTCTYCCACTTACTAT-3′, 5′-CACTTTCAAAAGAACAAGYGGATC-3′, 5′-TTGATCTCCCACATCATTGATG-3′, and 5′-CGAATATTCCAGYACTGAGAGAGTGG-3′ for polymerase basic 2 (PB2) gene segment, 5′-TGAAAGAAATCTCTGTGTCTTGTG-3′, 5′-CGAAAAGCTTGAACAGTCTGGGC-3′, 5′-TTGTATTCCCTCATGGTTTGGTGC-3′, and 5′-GAATCTTGGGCAAAAGAGATACAC-3′ for PB1 gene segment, 5′-TTCAAGGCTGGAGAAGTTCGG-3′, 5′-TCCTTTCGTCAGTCCGA-3′, 5′-GTTCCTGCTGATGGATGCTCTG-3′, 5′-ATCTTCTCCTATTTCATCAAGTTC-3′, and 5′-AACAGTATGACAGTGAYGAGCC-3′ for polymerase acidic (PA) gene segment, 5′-GAGGACATGCTGCCGTTACACC-3′,5′-GGAAATCCAGAGTGTGAATCACTC-3′, 5′-TGGACATTTTCCAATTGTGATCGG-3′, 5′-CAAAGTGAGGGATCAAGAAGGGAC-3′, 5′-CTGGCGACAGTTGAATAGATCGCC-3′, and 5′-GGACTACCACGATTCAAATGTG-3′ for HA gene segment, 5′- GGATGAGAGAACTCATCCTTTATG -3′, 5′-AAATGAGGTCTTCAATCTCAGCGTT-3′, 5′-ATGATCAAACGTGGAATCAATGAC-3′, and 5′-GTGGATGGCATGCCACTCTGCTGC-3′ for nucleoprotein (NP) gene segment and 5′-ATTRTCTTTACTGTATATAGCCC-3′, 5′-GCCACTCAATTCAACTTGGG-3′, 5′-CGACTGATTTGACTATCTTTCCC-3′, 5′-ATACAATGGCATAATAACAGACAC-3′, 5′-GCACCGTCTGGCCAAGACCAACC-3′, and 5′-GGGAGTTTTGTTCAGCATCCAG-3′ for NA gene segment.

### Animal experiments

Experiments were performed in 7- to 8-week-old female BALB/c mice (16–18 g; Charles River, Bad Sulzfeld, Germany). After isoflurane anesthesia, mice were inoculated intranasally with 10^6^ TCID_50_/20 µl of mpJena/5258 or HA-G222-mpJena/5258 diluted in EMEM. Mock-infected mice were used as control. Body weight and general condition was monitored for 12 (mpJena/5258) or 21 (HA-G222-mpJena/5258) days p.i. Mice that lost more than 25% of their initial body weight were sacrificed for humanity reasons. The general condition was evaluated with a laboratory clinical score from 0 to 7 as described recently [29].

Five to 10 mpJena/5258-infected mice were sacrificed on day 1–7, 9 and 12 p.i. Sera were obtained from the collected blood samples by centrifugation at 855× g for 15 minutes and stored at −20°C. The superior lobe of the lung and the trachea were aseptically removed, homogenized in test medium and titrated using MDCK cells for determination of TCID_50_ values. Another right lung lobe was frozen for RNA analysis. The left lobe was fixed in 10% formalin and embedded in paraffin. Sections of 6 µm were prepared and stained with hematoxylin and eosin.

### Neutralization assay

Sera of mice infected with mpJena/5258 were collected on day 1–7, 9, and 12 p.i. and used to determine antibody titers against HA-D222-mpJena/5258 and HA-G222-mpJena/5258. Furthermore, reactivity of HA-D222-mpJena/5258 and HA-G222-mpJena/5258 was analyzed with sera of HA-G222-mpJena/5258-infected mice obtained on day 21 p.i. Sera were inactivated at 56°C for 30 min. Two-fold serial dilutions of the sera in test medium were incubated with an equal volume of 100 TCID_50_ of the different test viruses for one hour at 37°C. Subsequently, 50 µl of these serum-virus mixtures were transferred on confluent MDCK cells covered with 100 µl test medium. Ninety-six hours later, cells were fixed and stained with crystal violet formalin solution. After removal of the dye and gentle washing, the assay was evaluated. The neutralizing antibody titer was defined as the reciprocal of the highest antibody dilution that prevented a cytopathic effect.

### Virus replication in MDCK and MDBK cells

Confluent MDCK and MDBK cells were infected at a multiplicity of infection (MOI) of 0.1 TCID_50_/cell of HA-D222-mpJena/5258 or HA-G222-mpJena/5258 and incubated at 37°C for one hour. Infection medium was removed and cells were washed three times. Afterwards, test medium was added and cells were incubated at 37°C. Supernatants were collected 1, 8, 24, and 48 hours p.i. and titrated using MDCK cells. The experiment was done two times with three parallels per time point.

### Detection of SAα2-3Gal and SA2-6Gal by lectin-binding assay

Confluent MDCK and MDBK cells were fixed with methanol. The expression of influenza receptors on the cell surface was analyzed as reported previously [34]. In brief, the digoxigenin (DIG)-labeled lectins *Maackia amurensis* (MAA) and *Sambucus nigra* agglutinin (SNA) of the DIG glycan differentiation kit (Roche, Mannheim, Germany) were used to detect SAα2-3Gal and SAα2-6Gal, respectively, according to the manufacturers protocol. For the red staining chromogenic solution of the DAKO REAL Detection System APAAP, Mouse was applied (Dako, Glostrup, Denmark).

### Red blood cell (RBC) binding and elution

The plaque-purified viruses HA-D222-mpJena/5258 and HA-G222-mpJena/5258 as well as each three trachea homogenates of virus-infected mice dissected on day 1 and 5 p.i. and once propagated on MDCK cells (mpJena/5258 trachea day 1 and day 5 p.i.) were studied for RBC binding and elution as described recently [35]. Two-fold serial virus dilutions were prepared in phosphate-buffered saline (171.1 mM NaCl, 3.3 mM KCl, 22.7 mM NaH_2_PO_4_ ×2 H_2_O, 1.8 mM KH_2_PO_4_) and incubated with an equal volume of a 1% suspension of chicken or sheep erythrocytes (both C-C pro, Oberdorla, Germany) in V-shaped 96-well plates (Greiner, Frickenhausen, Germany) at 4°C for one (chicken RBC) or two (sheep RBC) hours (RBC binding) followed by 37°C for one hour (RBC elution). The HA titer was defined as the reciprocal of the highest virus dilution that caused an agglutination of the erythrocytes.

### Statistical analysis

Statistical analysis was carried out with IBM SPSS Statistics 21. After confirmation of normal distribution, virus titers and antibody titers were compared by t test in case of equality of variances otherwise the Welch test was used. A probability value of<0.05 was considered as statistically significant.

## Results

### Biphasic course of disease is correlated with biphasic virus replication

After infection of BALB/c mice with three A(H1N1)pdm09 isolates, the isolate Jena/5258 caused severe influenza [29]. The disease was characterized by moderate body weight loss till day 3 p.i. followed by a short phase of recovery and further severe body weight loss till day 7 p.i., which was consistent with previous descriptions by other groups [11], [21], [28], [36]. To get a dynamic insight into the disease and to identify the driving force of the biphasic course of severe disease, the once mouse lung- and MDCK cell-passaged virus stock of Jena/5258 (mpJena/5258) was used to infect BALB/c mice. Sequencing of complete viral genomes verified that Jena/5258 and mpJena/5258 were identical (results not shown). After infection, mice were closely monitored over 12 days. Body weight changes, clinical score, lung and trachea virus titers, and lung histopathology were analyzed.

**Figure 1:**
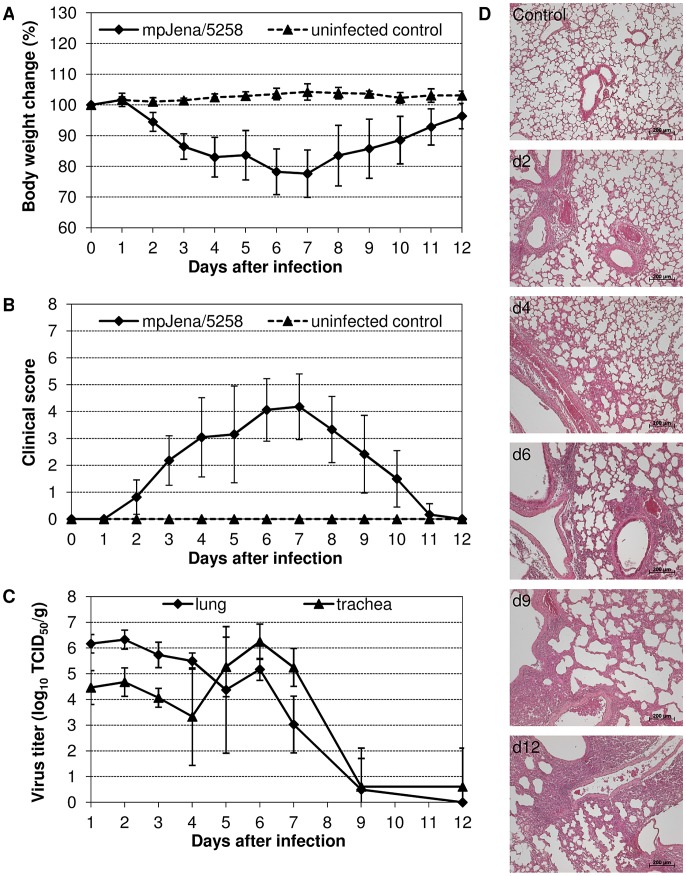
Course of infection induced by mpJena/5258 in BALB/c mice. (A) Body weight change and (B) clinical score of mice infected with 10^6^ TCID_50_ of mpJena/5258 and uninfected control mice were monitored for 12 days p.i. Mean body weight and standard deviations were calculated as percentage of initial body weight on day 0. (C) Mean virus titers in lung and in trachea as well as standard deviations of 5 (day 1–5), 6 (day 9 and 12) or 10 (day 6 and 7) infected mice on different days after infection were shown. (D) Hematoxylin-eosin stained lung tissue sections demonstrate the histopathological changes that were induced by virus replication on day 2, 4, 6, 9 and 12 after mp-Jena/5258-infection in comparison to an uninfected control mouse.

Like Jena/5258 [29], mpJena/5258 caused severe influenza. However, the severity of disease was markedly stronger. Mice came down with influenza on day 2 p.i. Continuous body weight loss was observed till day 3 or 4 p.i. On day 4 and/or 5 diseases stagnated in most animals. But, several mice lost more than 25% of their body weight and had to be mortified according to ethical rules on day 6 or 7 after infection (each ten mice). Comparison of individual curves of these sacrificed mice revealed that the dynamics of disease was mostly characterized by continuous worsening of symptoms (Fig. S1 and S2). The individual curves of remaining mice showed a biphasic course of disease (Fig. S1 and S2). In the result, the difference between the early and late stage of disease (mean body weight loss and clinical score) was less distinct (Fig. 1A, B) than that observed after infection with Jena/5258 recently [29].

Analysis of virus titers in lung and trachea revealed high values in both organs before symptom worsening (Fig. 1C). These results suggested that the symptoms were related to virus replication kinetics. Remarkably, the viral titer on day 2 p.i. was about 45 times higher in the lung than in trachea, whereas a 13 times higher virus titer was determined in the trachea than in the lung on day 6 p.i, which implied a change in the organ tropism of the virus in the course of infection.

The histopathological differences between the lungs of infected and uninfected mice were also analyzed (Fig. 1D). On day 2 p.i., infection resulted in acute bronchiolitis. In the following days, the surrounding lung tissue became inflamed. The number and size of infiltrates kept growing, despite the elimination of infectious virus at day 12 p.i. (Fig. 1C). At that time point more than 50% of the left lobe of the lungs developed infiltrates, edemas, and hemorrhages.

### HA-222D/G polymorphism determined organ tropism of mpJena/5258

**Figure 2:**
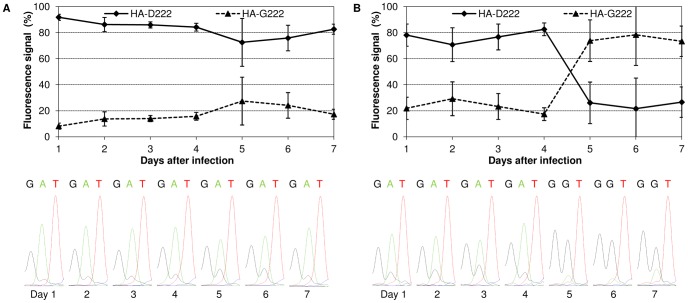
Proportion of HA-D222 and HA-G222 variant in lung and trachea during infection. The percentage of the fluorescence signal for the HA-D222 and HA-G222 variant in the sequence chromatogram of (A) lung (day 1–5: n = 5; day 6–7: n = 10) and (B) trachea (day 1–4: n = 5; day 5: n = 4; day 6–7: n = 9) samples was analyzed from day 1 to 7 p.i. The signal for different variants was measured for all mice individually. Mean values and standard deviations displayed in the figures were calculated from mice that were dissected on the same day. For each day a representative chromatogram of a trachea and a lung sample is shown. The nucleotides GAT code for the amino acid aspartic acid (D) and GGT for glycine (G).

To address the question whether the observed changes in organ tropism were caused by adaptive mutations in viral HA, total RNA was isolated from lung and trachea tissue homogenates from day 1 to 7 p.i. After reverse transcription with HA-specific primers, the HA gene segment was sequenced. Sequence analysis revealed a HA-222D/G polymorphism in lung and trachea samples. From samples collected on day 1 p.i., a strong adenine signal overlapped with a weak guanidine signal was observed at the second position of the coding triplet 222 (Fig. 2), which represents the amino acids aspartic acid (D) and glycine (G), respectively. This polymorphism was not detectable by Sanger sequencing in the HA gene sequence of the mpJena/5258 virus stock (results not shown), which was initially used in infection of BALB/c mice.

Further sequence analysis revealed that the proportion of HA-D222 and HA-G222 variant altered during the mouse infection experiment. An increase in fluorescence signal of guanidine for HA-G222 variant from 8% on day 1 p.i. up to 27% on day 5 p.i. was observed in the lung, whereas HA-D222 variant predominated (73–92%) during the whole experiment (Fig. 2A). Besides, HA-G222 variant represented the minor variant in trachea (17–29%) from day 1 to day 4 p.i., but became dominant after day 4 p.i. (Fig. 2B).

These results raised the question whether the HA-G222 variant was present in the trachea of mice infected with Jena/5258 [29]. Viral HA of each three lung and trachea tissue samples from Jena/5258-infected mice of day 4 p.i. (only available time point) was sequenced. As shown in Table 1, the HA-G222 variant was detected by Sanger sequencing in two of three trachea samples but, never in the lung.

**Table 1:** Proportion of HA-D222 and HA-G222 variant in lung and trachea tissue samples.

Tissue homogenate of	% of HA-222 variant in samples of			
	the lung		the trachea	
	HA-D222	HA-G222	HA-D222	HA-G222
Jena/5258-infected mouse 46^a^	100	n. d	n. d.	100
Jena/5258-infected mouse 47	100	n. d.	100	n. d.
Jena/5258-infected mouse 48	100	n. d.	9.9	90.1
HA-G222-mpJena/5258-infected mouse 27	n. d.	100	n. d.	100
HA-G222-mpJena/5258-infected mouse 28	n. d.	100	n. d.	100

a Lung homogenate of mouse 46 was used to get the mpJena/5258 virus stock.

n. d. not detected.

Taken together, these results demonstrated that the HA-222D/G quasispecies replicated with different efficiency in lung and trachea. The HA-G222 variant had a growth advantage in trachea, while the HA-D222 variant was predominating in the lung.

### HA-D222G affected receptor binding and HA-NA functional balance

Amino acid substitutions in HA might affect the receptor binding and/or the HA-NA functional balance, especially those within the receptor binding pocket, e.g. HA-222 [24]. To investigate the role residue HA-222 plays in receptor binding and in HA-NA functional balance, the replication of HA-D222-mpJena/5258 and HA-G222-mpJena/5258 was examined in cells with different receptor expression pattern. In addition, binding and elution of chicken and sheep red blood cells (RBC) by HA-222D/G quasispecies was compared.

HA-D222-mpJena/5258 was plaque-purified from the mpJena/5258 virus stock. HA-G222-mpJena/5258 was isolated from the trachea sample of an mpJena/5258-infected mouse dissected on day 5 p.i. Sequencing of HA-D222-mpJena/5258 and HA-G222-mpJena/5258 HA revealed an additional substitution HA-S84I in HA-G222-mpJena/5258. This position does not belong to the receptor binding or an antigenic site and presumably, would not affect receptor binding or antigenicity.

**Figure 3:**
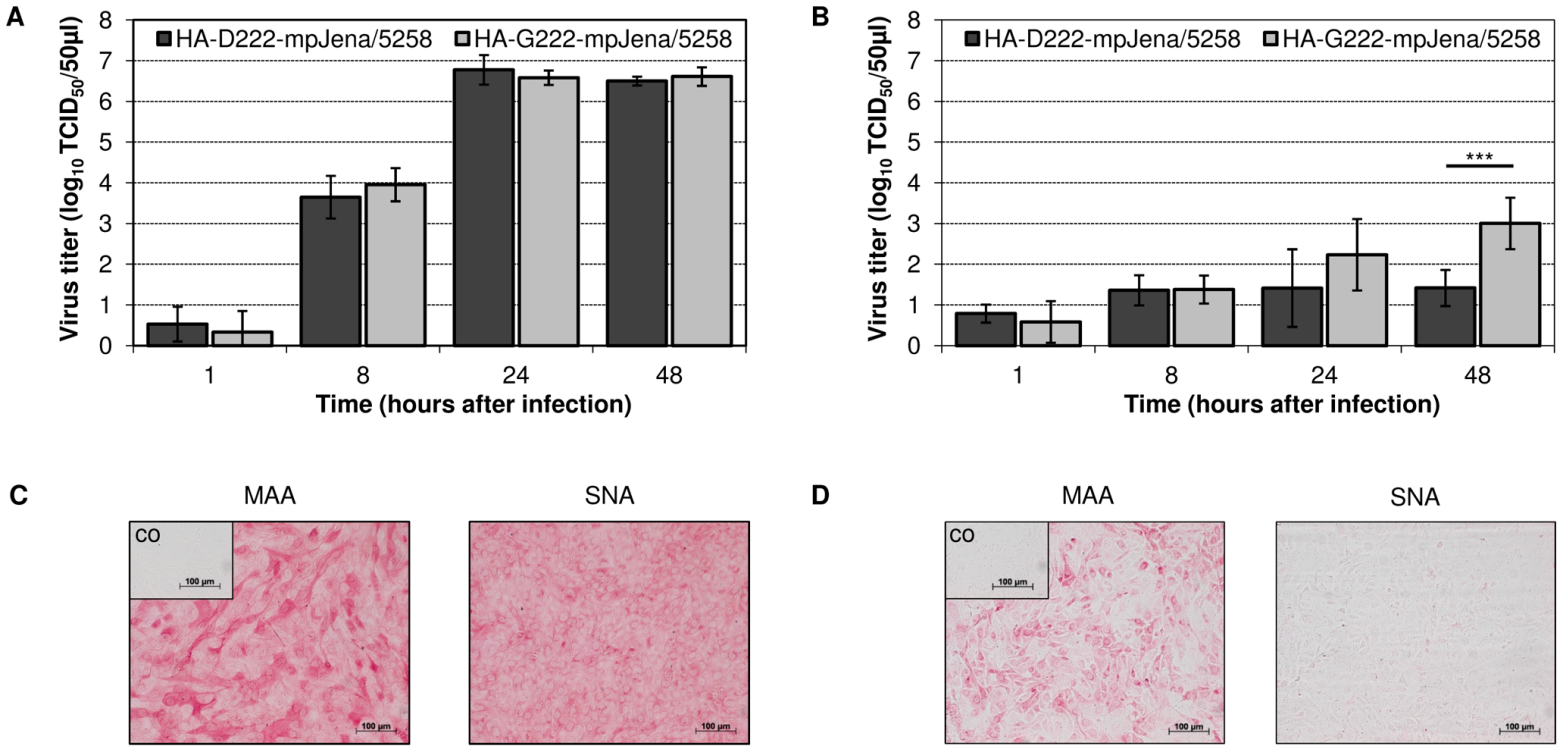
Replication of HA-D222-mpJena/5258 and HA-G222-mpJena/5258 and receptor distribution on MDCK and MDBK cells. (A) MDCK and (B) MDBK cells were infected with HA-D222-mpJena/5258 or HA-G222-mpJena/5258, respectively, at an MOI of 0.1 TCID_50_/cell. At the indicated time points supernatants were collected and virus titer was determined on MDCK cells. Mean values and standard deviations were calculated from 2 individual tests, each with 3 parallels. (C and D) Lectins MAA and SNA were used to show the distribution of influenza virus receptors SAα2-3Gal and SAα2-6Gal, respectively, in (C) MDCK and (D) MDBK cells. A lectin-free cell control (co) is included for both cell types. *** p<0.001.

Replication of HA-D222-mpJena/5258 and HA-G222-mpJena/5258 was compared in MDCK and MDBK cells. Differences in receptor expression pattern of both cell lines were demonstrated by immunohistochemical staining of these receptors with the lectins MAA for SAα2-3Gal and SNA for SAα2-6Gal, respectively. Large amounts of both viral receptors were detected on MDCK cell surface (Fig. 3C). In contrast, MDBK cells express almost exclusively SAα2-3Gal (Fig. 3D). HA-D222-mpJena/5258 as well as HA-G222-mpJena/5258 virus replicated much more efficiently in MDCK cells than in MDBK cells. Similar virus titers were determined for both virus variants in MDCK cells (Fig. 3A). However, in MDBK cells significantly higher titers were determined for HA-G222-mpJena/5258 than for HA-D222-mpJena/5258 at 48 hours p.i. (Fig. 3B). These results underlined a strong need of SAα2-6Gal for initiation of infection of both viruses and better recognition of SAα2-3Gal by the HA-G222 compared to the HA-D222 variant.

To confirm the receptor specificity, binding of HA-D222-mpJena/5258 and HA-G222-mpJena/5258 to chicken and sheep RBC at 4°C was analyzed. Chicken RBC express mainly SAα2-3Gal but also SAα2-6Gal and sheep RBC only SAα2-3Gal [27], [37]. Both plaque-purified virus variants caused hemagglutination of chicken RBC at 4°C. In contrast, only HA-G222-mpJena/5258 hemagglutinated sheep RBC (Table 2) confirming the binding affinity of the HA-G222 variant to SAα2-3Gal

**Table 2:** Differences in chicken and sheep RBC binding and elution by HA-G222 and HA-D222 variants.

Virus isolate	Hemagglutination titer			
	Chicken RBC		Sheep RBC	
	4°C	37°C	4°C	37°C
HA-D222-mpJena/5258	160	<10	<10	<10
HA-G222-mpJena/5258	80	80	40	<10
mpJena/5258 trachea day 1 p.i.^a^ (222D>>G)^b^	320	<10	<10	<10
	160	<10	<10	<10
	160	<10	<10	<10
mpJena/5258 trachea day 5 p.i.^a^ (222D<<G)^c^	160	80	40	<10
	160	40	40	<10
	80	40	20	<10

a Virus of trachea homogenates of three mpJena/5258-infected mice that were dissected on day 1 or 5 p.i. were propagated once in MDCK cells.

b On day 1 p.i. HA-D222 variant was the main species in the trachea.

c On day 5 p.i. HA-G222 variant was the main species in the trachea.

In addition, chicken as well as sheep RBC elution at 37°C within 1 hour was used to examine the HA-NA functional balance of HA-222G/D quasispecies. A good functional balance between viral HA and NA is represented by efficient elution of hemagglutinated RBC from virus [38]. Under these experimental conditions, complete elution from chicken RBC was observed for HA-D222-mpJena/5258 but not for HA-G222-mpJena/5258 (Table 2). In contrast, HA-G222-mpJena/5258 but not HA-D222-mpJena/5258 eluted from sheep RBC. Thus, substitution of amino acid HA-222 affected the HA-NA functional balance.

The results of RBC binding and elution were further confirmed by testing each three virus suspensions obtained from trachea homogenates of infected BALB/c mice dissected on day 1 and 5 p.i. On day 1 p.i., HA-D222 variant of mpJena/5258 predominated (HA-222D>>G; Fig. 2B). Therefore, these isolates behaved like HA-D222-mpJena/5258 (Table 2). The HA-G222 variant that became predominant in trachea on day 5 p.i. (HA-222D<<G; Fig. 2B) resembled RBC binding and elution properties of HA-G222-mpJena/5258.

### Increase of HA-G222 variant is favoured by weak neutralizing antibody response and/or recognition

**Figure 4:**
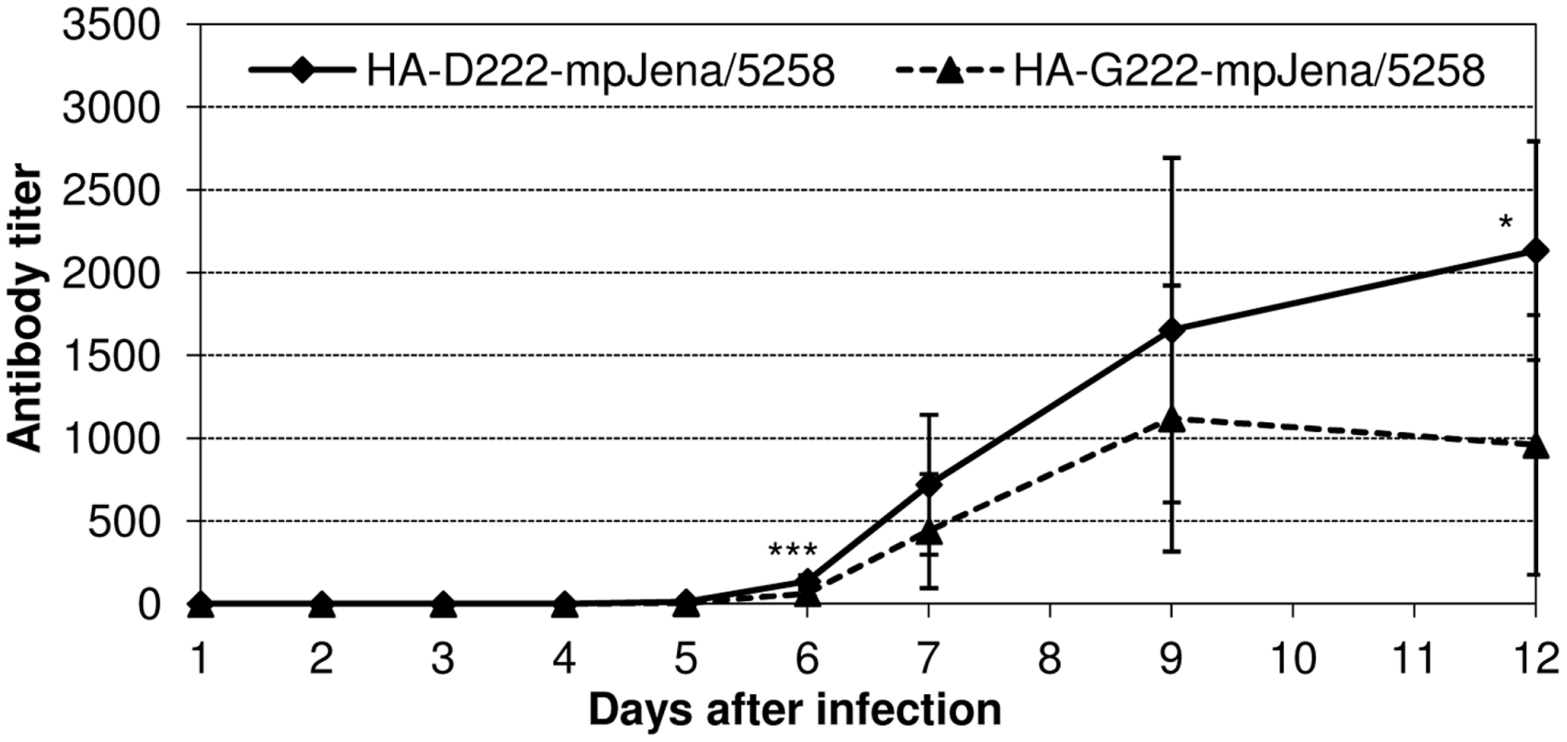
Detection of neutralizing antibodies. Sera of mpJena/5258-infected mice were tested with neutralization assay for HA-D222-mpJena/5258- and HA-G222-mpJena/5258-specific antibodies. Mean values and standard deviations were calculated from mice that were dissected on the same day. * p<0.05, *** p<0.001.

Because HA-222 is also a part of the Ca2 antigenic site [23], it was of interest to study the antibody response against both quasispecies. Sera of mpJena/5258-infected mice were collected on day 1–7, 9, and 12 p.i. and analyzed in a virus neutralization assay against HA-D222-mpJena/5258 and HA-G222-mpJena/5258, respectively, to see whether humoral immune response supported variant selection. Neutralizing antibodies against both quasispecies were detected in serum collected on day 6 p.i. The neutralizing antibody titer against HA-D222-mpJena/5258 was significant higher than that against HA-G222-mpJena/5258 (Fig. 4). On the following days anti-HA-D222-mpJena/5258 neutralizing antibody titers continued to be higher than anti-HA-G222-mpJena/5258 neutralizing antibody titers. These differences in variant-specific neutralizing antibody kinetics could result from the lower induction of antibodies due to the low proportion of HA-G222 variant in the first five days p.i. and/or from poorer recognition of HA-G222 variant by neutralizing antibodies.

To investigate if HA-G222 variant is generally more poorly recognized by neutralizing antibodies than HA-D222 variant, sera from mice that survived the infection with HA-G222-mpJena/5258 till the end of the experiment on day 21 p.i. were used to analyze cross-reactivity. Surprisingly, the test revealed a mean neutralization titer of 2011±684 for HA-D222-mpJena/5258, which was significantly higher than that of 1189±242 for HA-G222-mpJena/5258 (p = 0.018, results not shown). It implied a weaker recognition of HA-G222 variant by neutralizing antibodies.

### Plaque-purified HA-G222-Jena/5258 caused severe disease

To further confirm that the polymorphism of HA-222D/G has an impact on influenza dynamics, BALB/c mice were infected with 10^6^ TCID_50_ of plaque-purified HA-G222-mpJena/5258, the major variant in trachea in late infection.

**Figure 5:**
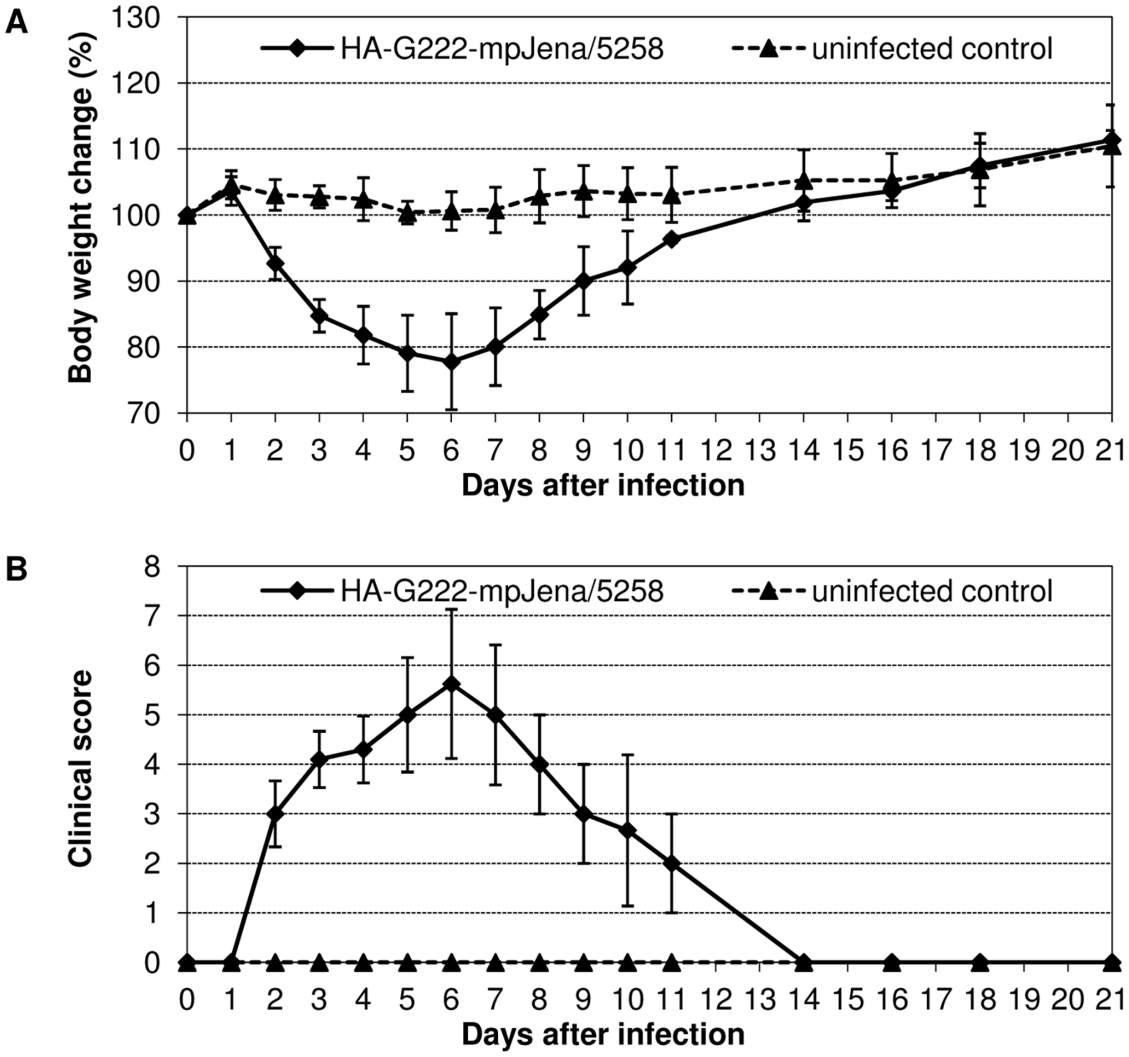
Course of disease after HA-G222-mpJena/5258 infection in BALB/c mice. (A) Body weight change and (B) clinical score of mice infected with 10^6^ TCID_50_ HA-G222-mpJena/5258 (n = 10) and uninfected control mice (n = 3) was monitored for 21 days p.i. Mean body weight changes and standard deviations were calculated as percentage of initial body weight on day 0.

Virus inoculation resulted in severe influenza with maximum mean body weight loss of 22.2% on day 6 p.i. (Fig. 5A). This maximum corresponded well to the severe body weight loss observed after infection of mice with mpJena/5258 at this time point (Fig. 1). In this experiment, 7 of 10 mice lost more than 25% of their body weight. Due to ethical considerations they were dissected between day 5 and 8 p.i. (results not shown) Remaining mice recovered and continuously gained body weight till the end of the experiment. The clinical score data were in agreement with the body weight kinetics (Fig. 5B). In the result of sequencing of viral HA of each two lung and trachea homogenates of day 4 p.i. only the HA-G222 variant was detected (Table 1).

These observations implicate that the severe symptom peak late in infection results from infection with the HA-G222 variant.

## Discussion

Using mpJena/5258 as example, the kinetics of replication of A(H1N1)pdm09 variants with HA-222D/G polymorphism was modeled for the first time *in vivo*. The results of the present study clarified the impact of HA-222D/G polymorphism on the severity and dynamics of influenza in BALB/c mice and identified factors that supported HA-222 quasispecies selection and influenza virus intra-host evolution.

As previously reported for human as well as for murine influenza [11], [18], [28], [29], [39], a severe and mostly biphasic course of disease was observed in the present study with mpJena/5258. Due to an increased severity of influenza observed in a portion of mice after infection with mpJena/5258, the peaks of the mean body weight loss and clinical score curves were less pronounced than that seen after infection with Jena/5258 [29]. Comparison of the percentage of HA-222D/G quasispecies in tissue samples of Jena/5258- and mpJena/5258-infected mice on day 4 p.i. demonstrated an accumulation of HA-G222 variant during mouse passage. The HA-G222 variant was shown to be highly pathogenic recently [15], [20], [21] as well as after infection of BALB/c mice with plaque-purified HA-G222-mpJena/5258 here. So, the accumulation of HA-G222 variant might explain the increased severity of disease observed in the present study.

Comparing the kinetics of symptoms (body weight changes and clinical score) with that of mpJena/5258 replication in lung and trachea of BALB/c mice, a correlation between viral load and symptoms was established. In addition, more efficient replication of mpJena/5258 in lung than in trachea in an early stage of infection on day 2 p.i. followed by dramatic increase in trachea virus titer and significantly higher viral load than in lung in the late phase of disease (day 6 p.i.) indicated the viral adaptation due to the change in organ tropism.

The HA-222D/G polymorphism identified by sequencing lung and trachea tissue isolates of infected mice between day 1 and 7 p.i. is in good agreement with recently published data [13], [40]–[42]. Interestingly, HA-D222 represented the major variant in the lung during the whole observation time. But, there was an increase of about 20% in the proportion of HA-G222 variant in lung over the time as described in a patient with severe A(H1N1)pdm09 infection recently [16]. In mouse trachea HA-G222 even became the major variant on day 5 p.i. So, the high mean virus titer in lung as well as in trachea on day 6 p.i. correlated with increased co-circulation of the HA-G222 variant. The role of HA-G222 variants and HA-222D/G polymorphism in severe influenza was discussed controversially until now [7]–[10], [12], [13]. The results of the present study clearly demonstrated that changes in the proportion of co-circulating quasispecies derived from HA-222D/G polymorphism were correlated with the shift in organ tropism.

The importance of quasispecies in viral evolution is well clarified [43]. For example, quasispecies enable antiviral drug escape [44] or enhance virus pathogenicity [45]. Here, an altered preference of HA-G222 variant to both SAα2-6Gal and SAα2-3Gal receptor facilitated changes in organ tropism of mpJena/5258 and promoted infection. Such alterations in receptor binding were also shown for other A(H1N1)pdm09 isolates [22], [25]. In addition, the elution of HA-G222-mpJena/5258 from chicken RBC (predominantly expressing SAα2-3Gal [37]) by NA was almost abolished, whereas HA-D222-mpJena/5258 could be efficiently released. In contrast, HA-G222-mpJena/5258 but not HA-D222-mpJena/5258 was eluted from sheep RBC. Whereas chicken RBC express only N-acetylneuraminic acid, sheep RBC present solely N-glycolylneuraminic acid [37]. Thus, the amino acid in HA-222 also affected the HA-NA functional balance that is important for viral release and spread.

The observed differences in receptor binding of HA-D222 and HA-G222 variant reflected the biphasic replication kinetics of mpJena/5258 in the upper and lower respiratory tract of BALB/c mice, as well. SAα2-3Gal is abundantly expressed on ciliated epithelial cells in the trachea of BALB/c mice, whereas SAα2-6Gal expression was sparing observed [46]. In mouse lung, SA2-3Gal is the main receptor on ciliated and non-ciliated cells of the bronchi and bronchioles. In addition, SAα2-6Gal was detected on pulmonary alveoli. The organ-specific receptor distribution pattern together with the differences in receptor binding of HA-D222 or HA-G222 variant resulted in better replication of HA-D222 variant in the lung and HA-G222 variant in the trachea of mice, respectively. It contributed to the evolutionary advantage of the HA-G222 variant in mouse trachea. In the past, three to ten lung-to-lung passages were required for mouse adaptation of A(H1N1)pdm09 viruses [17]–[20]. During lung-to-lung passages, tissue specimens were collected on day 4 p.i. or earlier [47]–[49]. However, according to the results of the present study the increase in the proportion of HA-G222 variant occurred in the lung after day 5 p.i. This gave rise to the difficulty in the isolation of HA-G222 variant and its detection by Sanger sequencing in the lung in the early stage of the disease after infection with Jena/5258. But, further accumulation of HA-G222 (7–27%) during the second lung passage enabled its detection. More sensitive next generation sequencing or pyrosequencing might be helpful to detect the subpopulations in circulating non-human viruses containing mutations and by this manner help to predict severe influenza.

A further factor contributing to the intra-host evolution of mpJena/5258 was the variant-specific humoral immune response. It was recently published that sera from A(H1N1)pdm09 virus infected and immunized people could provide variable cross-reactivity against several isolates possessing mutations in the antigenic sites [50]. HA-222 belongs to the antigenic site Ca2 [23]. The antibody response was lower against HA-G222-mpJena/5258 in comparison to HA-D222-mpJena/5258. It might result from the small proportion of HA-G222 variant in the early stage of infection similar to the effect from low-dose vaccinated mice [51]. However, significantly lower neutralization efficacy of sera of HA-G222-mpJena/5258-infected mice against HA-G222-mpJena/5258 in comparison to HA-D222-mpJena/5258 on day 21 p.i. could demonstrate a weaker neutralization of HA-G222 variant by antibodies. This might favor the replication of HA-G222 variant in the late phase of infection.

Taken together, the results obtained by studying the dynamics of mpJena/5258 infection in mice demonstrated that co-circulation of quasispecies derived from HA-222D/G polymorphism facilitates viral intra-host evolution. It is due to the dual function of this amino acid both as antigenic site and being part of the receptor recognition site. Despite known differences between receptor expression in the upper and lower respiratory tract of human and mice, our experiments visualized for the first time the dynamics of the intra-host evolution of A(H1N1)pdm09.

## Supporting Information

Figure S1 **Body weight change of individual mpJena/5258-infected mice.** Body weight change of individual mice infected with 10^6^ TCID_50_ of mpJena/5258 and uninfected control mice (mean) were monitored till the day of their dissection. Mice that were dissected on the same day are summarized in one diagram.
(TIF)

Figure S2 **Clinical score of individual mpJena/5258-infected mice.** Clinical score of individual mice infected with 10^6^ TCID_50_ of mpJena/5258 and uninfected control mice (mean) were monitored till the day of their dissection. Mice that were dissected on the same day are summarized in one diagram.
(TIF)
