# Supplementary figures and images for: Hemagglutinin 222D/G Polymorphism Facilitates Fast Intra-Host Evolution of Pandemic (H1N1) 2009 Influenza A Viruses
Source: PLoS One. 2014 Aug 27;9(8):e104233. doi: 10.1371/journal.pone.0104233 (PMC4146462; doi:10.1371/journal.pone.0104233)

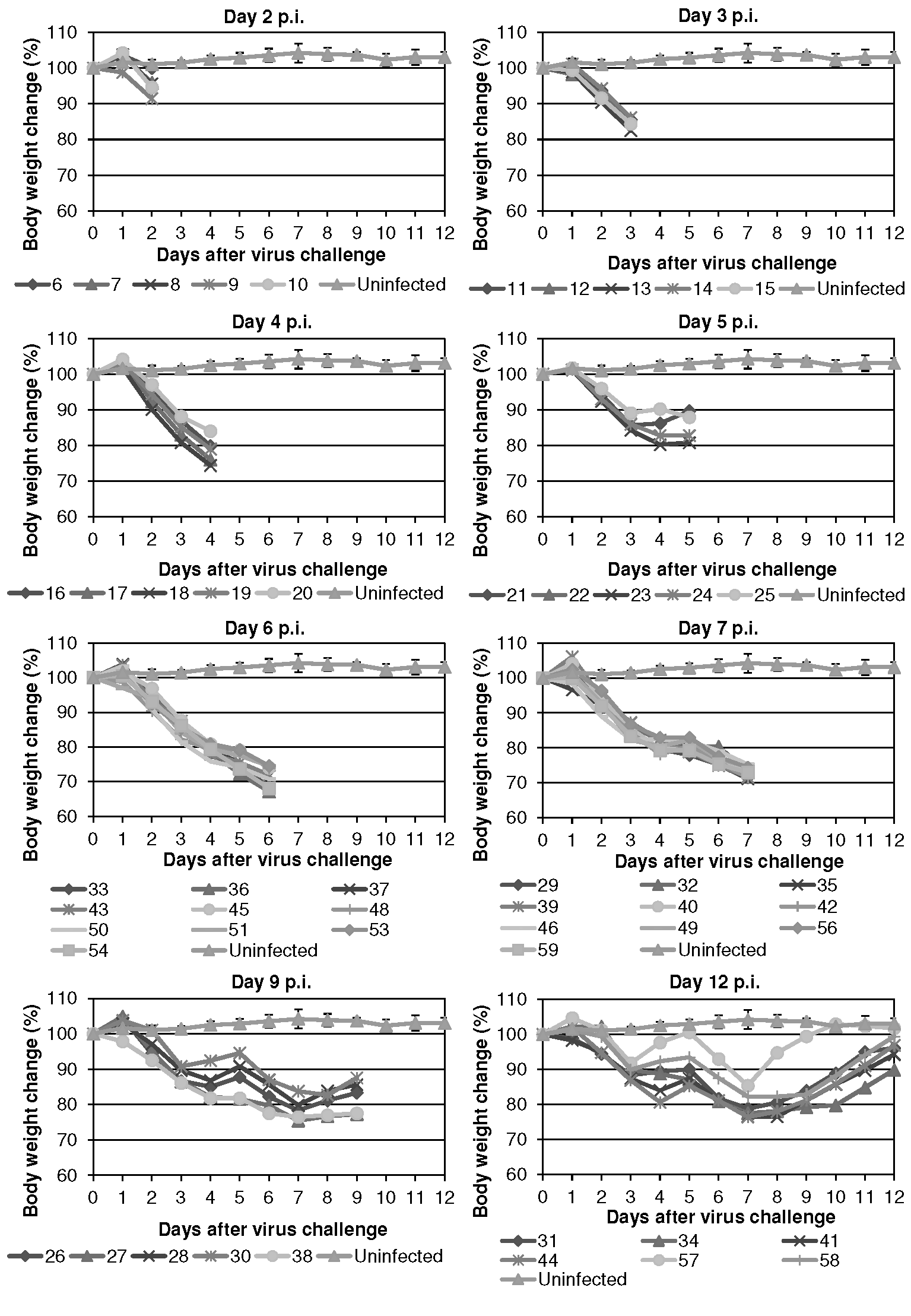

Supplement: Figure S1 — Body weight change of individual mpJena/5258-infected mice. Body weight change of individual mice infected with 106 TCID50 of mpJena/5258 and uninfected control mice (mean) were monitored till the day of their dissection. Mice that were dissected on the same day are summarized in one diagram. (TIF) [file pone.0104233.s001.tif]

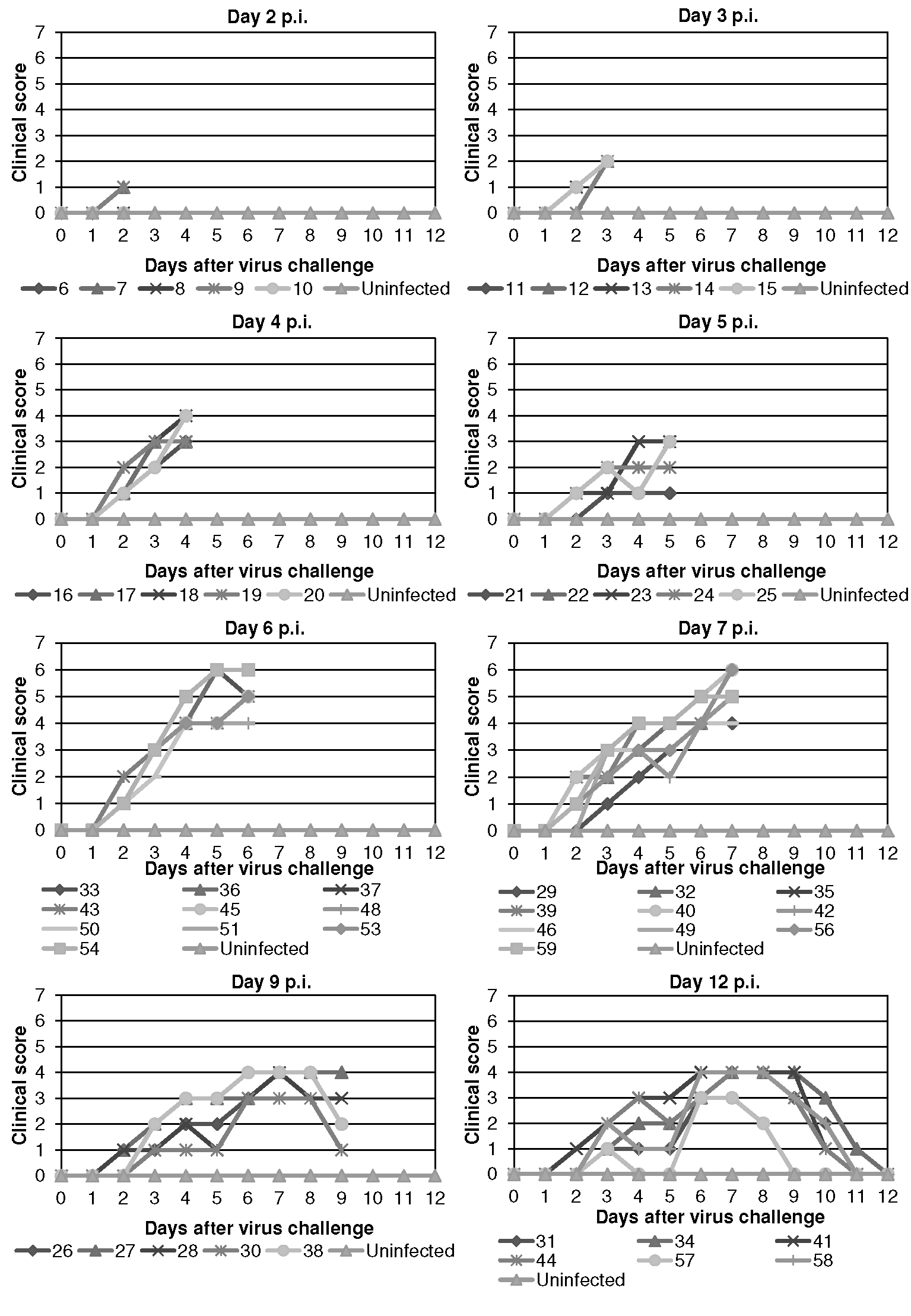

Supplement: Figure S2 — Clinical score of individual mpJena/5258-infected mice. Clinical score of individual mice infected with 106 TCID50 of mpJena/5258 and uninfected control mice (mean) were monitored till the day of their dissection. Mice that were dissected on the same day are summarized in one diagram. (TIF) [file pone.0104233.s002.tif]
